# Changes in the nutritional quality of products sold in university vending machines since implementation of the health star rating in 2014; an environmental audit

**DOI:** 10.1186/s12889-018-6177-z

**Published:** 2018-11-14

**Authors:** Yumeng Shi, Amanda Lee Grech, Margaret Allman-Farinelli

**Affiliations:** Nutrition and Dietetics Group, School of Life and Environmental Sciences, the Charles Perkins Centre, The University of Sydney, Level 4 East, Building D17, John Hopkins Drive, Camperdown, NSW 2006 Australia

**Keywords:** Young adults, Front-of-pack labelling, Nutrition, Diet, Public health, Tertiary institutions

## Abstract

**Background:**

Snacking is a prevalent dietary behaviour among young adults, which could independently contribute to weight gain. Vending machines provide easy access to unhealthy snacks and beverages for young adults in universities. A voluntary front-of-pack labelling, named the Health Star Rating (HSR) system, has been implemented nationally by the Australian government as one strategy to address obesity since 2014. The primary aim of this study was to detect changes in the availability, pricing and advertising of healthy and unhealthy snacks and beverages in university vending machines after introduction of the HSR.

**Methods:**

The study design was two cross-sectional audits of university vending machines one before (in 2014) and another after (in 2017) the implementation of the HSR. Data collections were conducted in a large urban university (> 60,000 students). Every machine was assessed; the product’s name, price, portion sizes and advertisements were recorded. Products were assigned an HSR to classify as healthy (≥3.5 stars) or unhealthy (< 3.5 stars). To compare the differences of product availability between 2014 and 2017, the Chi-square test was used.

**Results:**

A total of 1836 and 2458 slots were audited in 2014 and 2017, respectively. The proportion of healthy snacks and beverages increased from 7 to 14% (*p* < 0.001) and 38 to 44% (*p* < 0.05) since 2014, respectively. The mean costs of unhealthy snacks and healthy beverages increased after three years. Healthy food and drink options were more expensive than unhealthy choices in 2017. Advertisements on vending machines for unhealthy foods and drinks remained prevalent.

**Conclusion:**

Only small changes have been observed in the availability of healthy snacks and beverages in vending machines since implementation of the HSR system. Policy directives are indicated to encourage further improvements.

## Background

Young adults transition from adolescence to be responsible for their own lifestyles and dietary behaviours [1]. It has been shown that they consume more energy-dense snacks and sugar sweetened beverages, but less fruits and vegetables than other adults [1, 2]. These unhealthy food habits might be influenced by an obesogenic food environment, and could contribute to excessive weight gain and obesity during young adulthood [1, 2]. Many young adults can be reached in tertiary education institutions, thereby making this one appropriate setting to implement food environment interventions and nutrition programs to assist young adults with dietary behavioural change [3, 4]. As an example, in Australia, 1.7 million people aged 15 to 34 years old were studying for tertiary qualifications in 2016 [5].

Snacking is a prevalent dietary behaviour as a result of frequent meal skipping among young adults, which could independently contribute to weight gain [1, 6]. Vending machines provide easy access to snacks and beverages for younger adults to consume in the universities [7, 8]. Previous studies, including a vending machine audit in 2014 in an Australian university, found that the majority of the products in vending machines were unhealthy foods and beverages [9–11]. High availability of unhealthy options provides an environment that encourages the sales of unhealthy food products in preference to healthy foods [12].

The common variables of vending machine assessment include accessibility, availability, cost, promotion, and healthfulness criteria to categorise healthy and unhealthy products [7]. Interventions and policies to encourage healthier food choices from vending machines often target some of these variables [8]. Pricing and increasing the availability of healthier options have been identified as the most effective strategies to change consumer buying behaviours [8]. Several interventions have succeeded in increasing the sales of healthy snacks and beverages in university settings, e.g. setting discounts and addition of stickers for low-fat snacks, and labelling the products into different categories based on healthfulness [8].

In June 2014, the Australian and New Zealand governments introduced the Health Star Rating (HSR) which is a voluntary front-of-pack labeling system to assist consumers in comparing the nutritional quality of products in a quick and easy way [13]. Australia has one of the highest prevalences of obesity in the world and the HSR was implemented in response to the growing burden of obesity and weight-related lifestyle diseases [13]. The HSR ranges from half a star to five stars, with more stars indicating a healthier choice. Some state governments within Australia such as New South Wales have been setting benchmarks for availability of healthy food and drinks incorporating the HSR [14]. It is possible the introduction and more widespread adoption of the HSR could result in improvements in the products for sale including within vending machines and might assist better food choices.

The aim of this research was (i) to detect any positive changes in the availability of healthy food and beverage products in university vending machines after the nationwide implementation of the HSR system in Australia in 2014, (ii) to assess the compliance with recommended portion sizes and proportions of healthy (everyday) and unhealthy (occasional) products from the Healthy Food and Drink Framework by the State Ministry of Health [14], and (iii) to compare the cost and promotion of healthy and unhealthy foods between surveys.

## Methods

### Design

This study included two cross-sectional audits of vending machines within a large urban university (> 60,000 students) before and after the introduction of the HSR in Australia. The first vending machine audit was conducted between March and May 2014, and the second one was conducted between August and September 2017. Ethics approval was not required by the institutional Human Research Ethics Committee due to negligible risk of this study according to the National Statement on Ethical Conduct in Human Research [15].

### Measures

Vending machines on seven campuses of the selected university were evaluated. To conduct the assessment of vending machines, an audit tool from Kelly et al. [11] was used. Information was recorded on a standardized form for each slot of the vending machine including: the name, weight or volume and price of the product. Promotions on, inside or around the machine were also collected.

The nutritional quality of food and beverages was assessed according to the Australian government’s front-of-pack labeling HSR system [13]. The HSR is calculated based on nutritional composition which includes energy, positive nutrients (i.e. protein, dietary fibre and the proportion of fruit, vegetable, legume and nut content), and risk nutrients (i.e. saturated fat, sugar, sodium) with different criteria within food categories (Table 1) [13]. The steps to assess HSR for each food item and the criteria for each nutrient followed the Australian Government HSR Calculator guideline for industry [16].

**Table 1:** Food categories from the Health Star Rating^a^

Category 1	Beverages other than dairy beverages
Category 1D	Milk, dairy beverages (calcium content sufficient to make a source of calcium claim)
Category 2	All foods other than those included in Category 1, 1D, 2D, 3 or 3D
Category 2D	Dairy foods other than those included in Category 1D or 3D (includes yoghurts, cheeses with calcium content ≤320 mg/100 g; where mixed foods have ≤25% other non-dairy ingredients)
Category 3	Oils, oil spreads, margarine, butter
Category 3D	Cheese and processed cheese (with calcium content > 320 mg/100 g)

^a^From: http://www.healthstarrating.gov.au/

To further categorise the available foods and beverages as healthy and unhealthy, the criteria specified in the State Ministry of Health’s Healthy Food and Drink Framework were used [14]. Snacks with HSR of 3.5 stars and above were categorised as healthy options, while unhealthy snacks referred to the products with HSR below 3.5 stars. Plain water (allowed flavouring without added sugar and intense sweeteners), flavoured milk with an HSR of 3.5 stars and above, and 100% fruit juice were classified as healthy drinks by this Framework [14]. Unhealthy drinks referred to all kinds of sugar-sweetened beverages (e.g. soft drinks, energy drinks, flavoured mineral water and coconut water with added sugars) and diet drinks with artificial sweeteners that all rated 2 stars or below. The Framework suggests that the proportion of healthy (known as everyday) to unhealthy (known as occasional) food and drink choices should be 75% or more and 25% or less, respectively [14]. The Framework also includes portion size limits for specific foods and drinks in both healthy and unhealthy categories, e.g. energy-dense snack foods (no more than 50 g) and diet drinks (no more than 500 mL) [14].

### Analysis

Nutrient analysis of the foods and beverages was conducted based on the nutritional information panels and ingredient lists on the packages or online (e.g. the official website of the food company). Chi-square test was used to compare the difference between the proportions of products within each category in the vending machines in 2014 and 2017, while independent sample t-test was used to compare the cost difference between two audits. All statistical analysis was conducted in SPSS version 24.0, with *P*-values < 0.05 considered statistically significant.

## Results

### Sample

A total of 61 and 71 vending machines were recorded in the 2014 and 2017 audit, respectively. Snack machines (*n* = 27), beverage machines (*n* = 33), and the machines that contained both snacks and beverages (*n* = 11) were found in seven campuses of the university in the recent audit. In total, 1836 and 2458 occupied slots were recorded in 2014 and 2017, respectively. One ‘Veggie Vendors’ machine was excluded from the analysis because it was not part of the active vending system across the campuses. It had been installed on a remote campus as a trial by the Healthy University Committee.

### Availability

Of snack slots, 14% (157 out of 1158 slots) were rated as 3.5 stars and above in 2017, which doubled the percentage (7%) from the previous audit (*p* < 0.001). Regarding beverage slots, 44% (578 out of 1300 slots) were categorised as 3.5 stars and above, which was 6% higher from 2014 (*p* < 0.05). On the other hand, the total number of slots with unhealthy choices also showed an increase from 847 to 1001 slots in snacks and 570 to 722 slots in beverages, but the percentage increase of healthy snack and beverage slots was greater. The proportions of the products that ranged from 5 stars to 0.5 stars in the two audits are listed in Table 2 (snacks) and Table 3 (beverages).

**Table 2:** Proportion of snacks available in university vending machines with different Health Star Rating^a^

HSR (stars)	Slots % (*n*)		*P* value
	2014 (*n* = 910)	2017 (*n* = 1158)	
*Healthy*			
5	0 (1)	2 (28)	< 0.001
4.5	2 (22)	3 (39)	0.205
4	0 (0)	4 (41)	< 0.001
3.5	4 (40)	4 (49)	0.855
Total	7 (63)	14 (157)	< 0.001
*Unhealthy*			
3	4 (34)	6 (68)	0.026
2.5	23 (208)	23 (270)	0.806
2	10 (87)	8 (95)	0.280
1.5	7 (67)	6 (75)	0.429
1	11 (100)	13 (153)	0.126
0.5	39 (351)	29 (340)	< 0.001
Total	93 (847)	86 (1001)	< 0.001

^a^http://www.healthstarrating.gov.au/

**Table 3:** Proportion of beverages available in university vending machines with different Health Star Rating^a^

HSR (stars)	Slots % (n)		*P* value
	2014 (*n* = 926)	2017 (*n* = 1300)	
*Healthy*			
5	36 (330)	33 (433)	0.254
4.5	0 (0)	2 (28)	< 0.001
4	0 (0)	2 (25)	< 0.001
3.5	3 (26)	7 (92)	< 0.001
Total	38 (356)	44 (578)	0.005
*Unhealthy*			
3	0 (0)	3 (44)	< 0.001
2.5	0 (0)	1 (10)	0.007
2	12 (115)	17 (216)	0.006
1.5	9 (82)	10 (129)	0.397
1	40 (373)	22 (289)	< 0.001
0.5	0 (0)	3 (34)	< 0.001
Total	62 (570)	56 (722)	0.005

^a^http://www.healthstarrating.gov.au/

Snacks rated 3.5 stars and above numbered 10 in 2014 and 35 products in 2017. These included vegetable chips, legumes and nuts, cheese or tuna with crackers, and ready-to-eat meals with small portions. Chips and other snacks made from the extrusion of cereal flours or starches (mostly with an HSR of 2.5 stars) remained as the most prevalent unhealthy snacks (37%), while chocolate (mostly with an HSR of 0.5 stars) comprised the second largest proportion of unhealthy snacks (26%). Nevertheless, snacks with an HSR of 0.5 stars decreased 10% since the previous audit.

For beverage options, more varieties of both healthy and unhealthy were seen in 2017. Water was the most frequently available healthy drink occupying 27% of all beverage slots, followed by dairy beverages (14%) and 100% fruit juice (4%). Those sugar-sweetened beverages with a HSR of 1 star (e.g. coke and energy drinks) were still the most common unhealthy beverage products, but occupied 18% less of beverages slots compared with 2014. Beverages with HSR between 2 and 4.5 stars (e.g. diet beverages and flavoured milk) substituted for the 1 star drinks showing significant increases after 3 years.

The majority of vending machines (92%) contained at least one option rated 3.5 stars or above. On average, each snack machine had three healthy choices with a mean proportion of 9%, while 40% (*n* = 14) of beverage machine contents were healthy. Most vending machines (*n* = 64) are supplied by one contractor with the university food service operator and were present in the 2014 audit. The product availability in these vending machines remained unchanged after 3 years. After the 2014 audit, a total of seven machines from a new vending company had been introduced in four libraries and three teaching buildings by university staff rather than the food service providers. These new vending machines had a higher proportion of snack (41%) and beverage (70%) options rated 3.5 stars and above.

Only 26 of 301 snack and beverage products (8.6%) had the HSR classification labelling on their packages in 2017. Of these, 24 items had 3.5 stars or above, and another two had 3 stars. Within the machines introduced by the new vending company, 15 out of 69 available products (22%) carried a HSR label on the snack and beverage packages.

### Portion size

The average portion sizes and the percentages meeting recommended portion limits within several popular categories of snacks and beverages in the 2017 audit are listed in Table 4. Within the snack categories shown in Table 4, the portions of a large proportion of snacks exceeded the recommended portion sizes, except for chips. Regarding beverages, the mean portion sizes of soft drink and diet beverages were more than the recommended 500 mL, but the other drinks (i.e. dairy beverages, energy drink and juice) were typically served in smaller containers.

**Table 4:** Comparison of the portion sizes of snacks and beverages with recommended portion limits

Food categories (*n*)	Mean portion size (SD)	Within portion limit (%)	Maximum portion limit^a^
*Snacks*			
Confectionery (92)	113 g (85)	52%	50 g
Sweet Biscuits (21)	90 g (2)	0%	50 g
Muesli/nut bars (119)	59 g (22)	40%	50 g
Nuts/legume snacks (51)	61 g (19)	65%	50 g
Chocolate (298)	52 g (17)	67%	50 g
Chips/extruded snacks^b^ (490)	45 g (8)	95%	50 g
*Beverages*			
Water (355)	571 ml (65)	Not applicable	No restriction
Diet beverages (170)	551 ml (105)	19%	500 ml
Soft drinks (393)	507 ml (109)	Not applicable	Should not be sold
Dairy beverages (208)	468 ml (66)	100%	500 ml
Energy/sports drinks (127)	412 ml (138)	Not applicable	Should not be sold
Fruit juice (47)	354 ml (33)	96%	400 ml

^a^Maximum portion size limits listed in the Healthy Food and Drink Framework by the State Ministry of Health

^b^Extruded snacks: snacks made from the extrusion of cereal flours or starches

### Cost

The mean costs of healthy and unhealthy products in both audits are shown in Table 5.

**Table 5:** The mean costs of healthy vs. unhealthy products in 2014 and 2017

Food Categories	Mean (SD)		*P* value
	2014	2017	
*Snacks*			
Healthy	$2.74 (0.79)	$3.27 (1.12)	0.169
Unhealthy	$2.43 (0.44)	$2.86 (0.89)	< 0.001
*Beverages*			
Healthy	$3.25 (0.45)	$3.77 (0.89)	0.006
Unhealthy	$3.63 (0.62)	$3.36 (0.86)	0.119

Between 2014 and 2017, there was a significant increase of $0.43 in the cost of unhealthy snacks (*p* < 0.001), while the cost of healthy snacks rose by $0.53 (*p* = 0.169). The average cost of healthy beverages increased by $0.52 (*p* = 0.006), but the drop of $0.27 in the cost of unhealthy drinks between the two time points was not significant. Healthy snacks (*p* = 0.05) and beverages (*p* = 0.009) were more expensive than unhealthy choices in 2017.

### Promotion

The majority of vending machines contained advertisements on or inside the machines. Of snack machines, 81% (*n* = 22) had promotions for unhealthy snacks (e.g. potato chips) in 2017 compared to 71% (*n* = 20) in 2014 (*p* > 0.05), with no advertisements on the remaining machines. Of beverage machines, the proportion of advertisements for healthy drinks increased from 52% (*n* = 14 for water only) to 62% (*n* = 16 and 8 for water and dairy beverages, respectively) after 3 years (*p* > 0.05). On the other hand, the proportion of unhealthy beverages advertisements (e.g. soft drinks and energy drinks) decreased from 48% (*n* = 13) to 38% (*n* = 15). Amongst 11 machines that contained both snacks and beverages in the recent audit, four had non-specific promotion (i.e. no specific brand was shown) and the remaining were the vending machines supplied by the new vending company.

This new vending company claimed their machines contained ‘zero junk food’ and encouraged consumers to ‘snack with confidence’. Different promotion strategies were utilized including discounts and digital advertisements for healthy options, such as nut bars and dairy products. Moreover, they provided nutritional labelling at the point-of-purchase, such as labels for protein sources and nuts contents next to the slot number inside the machine. On the digital screen, a display of products with no added sugar, gluten free, dairy free and protein sources were also available for consumers.

Overall, 67% (*n* = 22) of beverage machines provided a ‘Kilojoules count’ table which was introduced by the beverage company. It displayed the portion sizes and the energy values of drinks from their company for customers to compare.

## Discussion

The availability of healthy snacks and beverages in the university vending machines improved since the introduction of the HSR in 2014. However, although more varieties of foods and drinks with better nutritional quality were detected in the most recent audit, the proportions of healthy to unhealthy choices were still well below the benchmarks from the State Ministry of Health’s Healthy Food and Drink Framework. In addition, the cost of healthy choices was not yet competitive with unhealthy options in 2017 and promotions for unhealthy choices were prevalent, particularly concerning discretionary snacks.

There was increased variety and availability of snacks and beverages with the HSR of 3.5 stars and above in 2017, but within individual snack vending machines, the majority contained few healthy choices. The exceptions were the seven machines from the new vending company that contained a higher proportion of healthy snacks, but also provided the majority of the snacks with HSR of 3.5 stars and above available in the vending machines on campus. They stocked most of the products that carried the HSR label on their packaging. An actual increase in the number of slots in the vending machines meant the vending system in 2017 provided more access to snacks and drinks, both healthy and unhealthy, for university students compared with 2014.

Since implementation of the voluntary HSR system in 2014, uptake of the system on products sold at four major New Zealand supermarkets has been slow, and it is estimated only 5% of products display the stars on their packages [17]. There was evidence that the HSR system influenced product reformulation and the products that displayed the system on their packages, had a more favourable nutrient profile than before the system was implemented [17]. In the vending machines audited, 8.6% of products displayed the stars in 2017 and of the 26 products displaying it, 24 had 3.5 stars or more. Thus as was found in supermarkets only those with favourable profile carry the stars. Some food companies reported challenges in the implementation of the HSR system, such as difficulties in HSR calculation and additional costs on packaging and human resources as reasons for not implementing it [18]. The authors had no difficulty applying the instructions provided by the Australian Government HSR Calculator guidelines for industry. Clearly the scheme will fail to achieve its aim of improving food choices by the public unless it is widely adopted. Mandatory regulation on the HSR system would appear to be needed.

Providing healthier options can change consumer sales towards healthy foods [8, 19]. Consumers have already expressed interest in salads, fresh fruit, and yogurt [20, 21]. Foods with shorter shelf-life require refrigeration but this is no longer the technical obstacle in storing snacks that it was previously [19]. The difficulties in finding products with long shelf-life, appropriate package size and competitive prices for vending systems [22] are becoming less. There are an increasing number of vending companies that provide healthy options both in Australia and USA [23, 24]. Consequently, it is recommended these types of vending companies replace the current contractor’s machines which contained 90% unhealthy choices. For example, if the university food service operator contracted solely the vending machine company that supplied the seven new machines, then healthy options would comprise 40% of vending machine contents, which is a step closer to the 75% the framework recommended [14].

Portion size is not considered in the HSR system, but products with 3.5 stars and above should not be consumed without restriction. Large portion sizes have been implicated in the obesity epidemic [25, 26]. Therefore, the State Ministry of Health’s Framework provides portion limits for several energy-dense food categories, such as nuts and chocolate [14]. Diet beverages failed to meet the recommended volumes. Substituting these drinks with smaller containers might be a feasible intervention [22]. The same strategy could also be the first step to gradually prohibit sugar-sweetened beverages in the university vending system. Unhealthy snacks need to be served in smaller packages as well, but a group of interventions should be implemented simultaneously to prevent increases in purchases which might be stimulated by smaller portions [11].

Cost has been found to be an important factor that affects food choices from vending machines, particularly when purchasing healthier food items [27]. In Mexico, a two-year sustained reduction of sugar-sweetened beverage consumption was found as a result of taxation [28]. The World Health Organization has called for taxes on sugar-sweetened beverages to be implemented internationally [29]. To encourage more purchases of healthy options, price discounts may attract more university students to change their food choices. A study from the USA indicated that a price reduction of 10% was able to assist consumers in making better food choices without compromising the profits [30]. Slightly increasing the price of unhealthy options simultaneously was suggested as another potential strategy to expand the price gap and maintain the revenues [30].

There is an urgent need to protect young adults who are susceptible and frequently targeted by food companies’ marketing but are quite often forgotten in public health initiatives [31]. The proportion of advertisements on unhealthy items remained high, especially for snacks. Although the new vending company machines contained promotions for healthy options, they also claimed to contain no junk foods. However, our study demonstrated that more than half of the snacks offered had a HSR below 3.5 stars. Promotions for unhealthy foods and drinks are not permitted according to the Framework for the State government health facilities and schools [14, 32]. However, these policies are not in place in universities and the onus is on universities to set their own policies targeting young adults who are vulnerable to food promotions [31].

Providing nutritional labelling at the point-of-purchase has been utilized as a part of intervention strategies in many studies [8]. The design of vending machines can make the original nutritional information on the packages invisible for customers [33], and thus extra labelling on or inside the machines might make it easier for them to choose healthier products. The stickers for protein source and nuts in the new machines were good examples, and more should be incorporated into the whole vending system. Stickers for the slots of those snacks and drinks with 3.5 stars and above could be a feasible method.

A combination of strategies has been suggested as the most cost-effective way to create a food environment which makes healthy food choices easier for consumers [11, 34]. This may include increasing the availability and reducing the cost of healthy options, forbidding promotions on unhealthy foods and decreasing their portion sizes. Additionally, it is extremely important to engage stakeholders, such as vending companies, decision-makers in the universities and students [35]. To guarantee the sustainable effect of any such interventions, follow-up studies are necessary to evaluate the compliance and adjust the plan accordingly [36].

This study is the first one to apply the HSR system to assess food products in vending machines in an Australian university. The two time points selected allowed detection of changes before and 3 years after implementation of the HSR system. A strength of the study is that all vending machine data was collected by researchers in audits, rather than reports from the vending company. Moreover, the calculations of the HSR for every product were verified by using the HSR Calculator on the official government website. The results from this study are generalizable for other Australian universities which are known to use the same primary vending suppliers as a common organization facilitates the contractual agreement for almost all tertiary education institutions in Australia.

One of the limitations of the current study is the lack of sales data, and it is unknown if sales of healthy versus unhealthy items has increased. During the process of calculating the HSR, there were some estimations for the proportions of fruits, vegetables, nuts and legumes that had to be made due to insufficient details from the ingredient lists. A registered dietitian was responsible for this analysis and the overall results of these products are not expected to be significantly affected. Ideally, setting up a control group would facilitate the demonstration of internal validity, but the HSR system was implemented simultaneously across the country which prohibited this. However, the limited effect of the current policy was clearly indicated by our findings.

## Conclusion

Healthy snacks and beverages have become more available in university vending machines in 2017 compared to 2014, but fell well short of the Framework to achieve a healthy food environment. The introduction of the voluntary HSR food labeling system failed to provide substantial improvements in the nutritional quality of products offered and few products carried the label on their packaging. Some progress has been made in beverages offered, but healthy snacks were more available only because of the introduction of some new vending machines by a small company interested in selling healthy products. Machines operated by the largest suppliers for Australian universities remained unchanged. Policy directives may be necessary for further improvements in the availability of healthy foods and drinks with competitive pricing and better promotions, to facilitate healthier food choices amongst young adults in tertiary education institutions. Regulation rather than a voluntary system for displaying HSR would appear to be necessary.

## Abbreviations

HSR: Health Star Rating
